# Data of clavulanic acid and clavulanate-imidazole stability at low temperatures

**DOI:** 10.1016/j.dib.2019.103775

**Published:** 2019-03-07

**Authors:** David Gómez-Ríos, Howard Ramírez-Malule, Peter Neubauer, Stefan Junne, Rigoberto Ríos-Estepa

**Affiliations:** a Universidad de Antioquia, Departamento de Ingeniería Química, Grupo de Bioprocesos, Calle 70 No. 52-21, Medellín 050010, Colombia; b Universidad del Valle, Escuela de Ingeniería Química, A.A. 25360, Cali 76001, Colombia; c Technische Universität Berlin, Department of Biotechnology, Bioprocess Engineering, Ackerstr. 76, ACK 24, Berlin 13355, Germany

## Abstract

Clavulanic acid (CA) is a β-lactam antibiotic with a strong inhibitory effect on β-lactamase enzymes. CA is produced in submerged cultures by the filamentous Gram-positive bacterium *Streptomyces clavuligerus (S. clavuligerus).* CA is an unstable molecule in aqueous solution and its stability depends strongly on temperature and concentration. In this contribution, the experimental data of CA stability, produced in chemically defined media and exposed to temperatures between −80 and 25 °C, are presented. The chromophore clavulanate-imidazole (CAI) is commonly used for analysis and quantification of CA samples by High Performance Liquid Chromatography (HPLC); nevertheless, this molecule is also susceptible to suffer degradation in aqueous solution, potentially affecting the quantification of CA. Data of CAI concentration for samples conserved at 4 °C and 25 °C are also presented. A reversible-irreversible kinetic model was applied to estimate the degradation rate of CA. Data from numerical simulations of CA degradation using the proposed kinetic model are also graphically presented. The data show the clavulanic acid instability in fermentation broths, in a range of temperatures of interest for bioprocess operation, downstream processing, samples quantification, conservation and storage.

**Table:** Specifications table

Subject area	*Biotechnology*
More specific subject area	*Antibiotics stability and Antibiotics analysis*
Type of data	*Table, equation, figure*
How data was acquired	Analysis of clavulanic acid concentration by HPLC with a DAD detector (1200 Series, Agilent Technologies, Waldbronn, Germany) and a Zorbax Eclipse XDB-C-18 chromatographic column with a C-18 guard column [1]
Data format	Processed, simulated
Experimental factors	Supernatant samples (undiluted, diluted 1:2 and 1:5) from *S. clavuligerus* batch fermentations were stored at −80 °C, −20 °C, 4 °C and 25 °C for analysis of clavulanic acid at different times in a time span of 43 h. Additionally, chromophore clavulanate-imidazole samples (0.636 mM and 0.310 mM) were stored at 4 °C and 25 °C and their concentration were measured at different times, up to 46 h.
Experimental features	Kinetics of degradation of clavulanic acid from fermentation broths and at different initial concentrations was determined at low temperatures of storage and pH 6.8, using a factorial experimental design. Kinetics of degradation of the chromophore clavulanate-imidazole with different initial concentrations was calculated with a 2-squared factorial design. Linear regression was applied to the data for the determination of kinetic parameters.
Data source location	Technische Universität Berlin, Institute of Biotechnology, Chair of Bioprocess Engineering. Berlin, Germany.
Data accessibility	Data are presented in this article only
Related research article	D. Gómez-Ríos, H. Ramírez-Malule, P. Neubauer, S. Junne, R. Ríos-Estepa, Degradation Kinetics of Clavulanic Acid in Fermentation Broths at Low Temperatures, Antibiotics. 8 (2019) 6. https://doi.org/10.3390/antibiotics8010006.

**Table:** 

**Value of the data** • Data show the clavulanic acid instability in fermentation broths, in a range of temperatures of interest for bioprocess operation, downstream processing, samples quantification, conservation and storage. • Data of instability of the chromophore clavulanate-imidazole are of utmost importance in the analytical field, since quantification of clavulanic acid must consider the time elapsed between sample collection, sample derivatization and quantification. • The basic information about thermal and concentration effects on clavulanic acid degradation kinetics are required to understand the instability of the clavulanic acid molecule in aqueous solutions and their potential reaction mechanisms. • The applied kinetic model of degradation and simulation allow to predict the degradation of clavulanic acid at different concentrations and temperatures. • The obtained data can be useful for further comparative initiatives of exploring clavulanic acid stability under different conditions.

## 1 Data

**Fig. 1:**
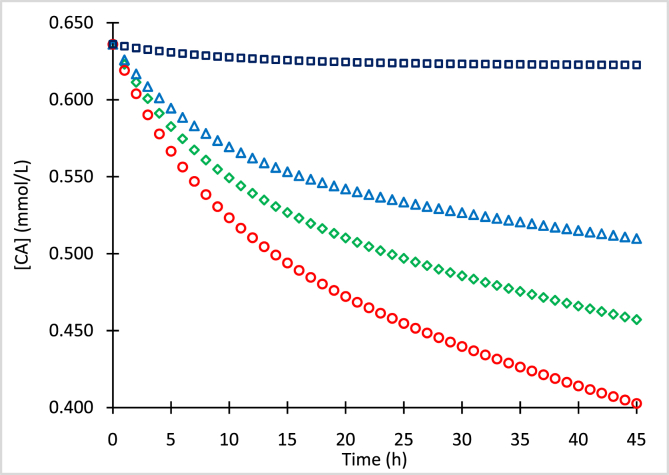
Simulation of CA degradation of CA_o_ = 0.636 mmol/L at −80 °C (squares), −20 °C (triangles), 4 °C (diamonds) and 25 °C (circles).

**Fig. 2:**
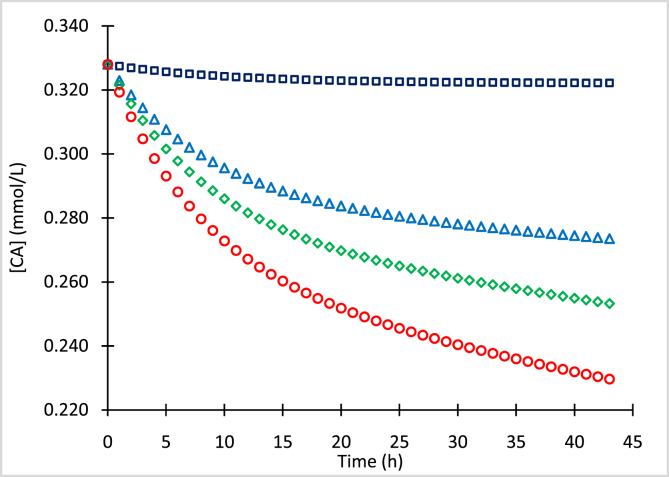
Simulation of CA degradation of CA_o_ = 0.329 mmol/L at −80 °C (squares), −20 °C (triangles), 4 °C (diamonds) and 25 °C (circles).

**Fig. 3:**
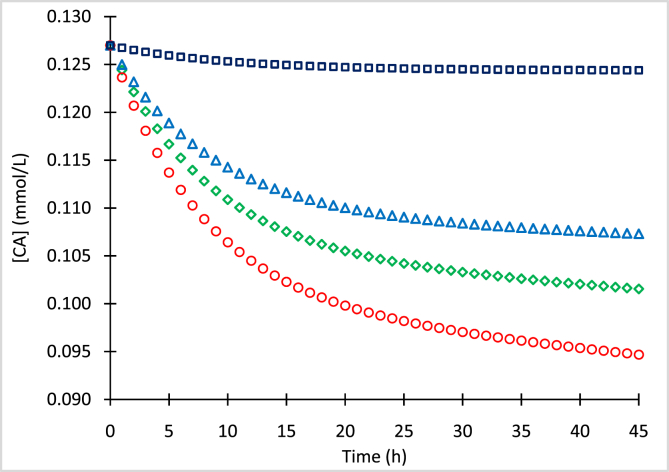
Simulation of CA degradation of CA_o_ = 0.127 mmol/L at −80 °C (squares), −20 °C (triangles), 4 °C (diamonds) and 25 °C (circles).

**Fig. 4:**
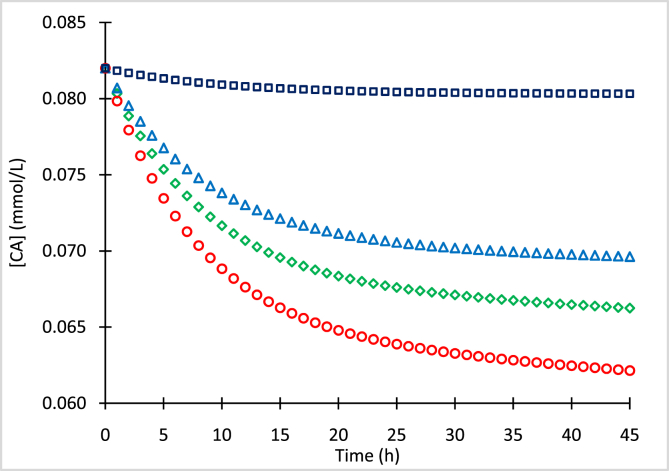
Simulation of CA degradation of CA_o_ = 0.082 mmol/L at −80 °C (squares), −20 °C (triangles), 4 °C (diamonds) and 25 °C (circles).

The mean concentrations of clavulanic acid (CA) and standard deviations (SD) for processed samples with initial concentrations (CA_o_) of 0.636 mmol/L (Table 1), 0.329 mmol/L (Table 2), 0.127 mmol/L (Table 3), and 0.082 mmol/L (Table 4) stored at −80, −20, 4 and 25 °C, are presented. Concentration data of the chromophore clavulanate-imidazole (CAI) with initial concentrations (CAI_o_) of 0.310 mmol/L and 0.636 mmol/L, conserved at 4 °C and 25 °C, are presented in Table 5, Table 6, respectively. Fig. 1, Fig. 2, Fig. 3, Fig. 4 show numerical simulation data of the equilibrium-irreversible first-order kinetic model used to describe the degradation of CA samples.

**Table 1:** Mean CA concentrations for samples of CA_o_ = 0.636 mmol/L (126.7 mg/L) conserved at −80, −20, 4 and 25 °C, and quantified at different times.

Time (h)	T = −80 °C		T = −20 °C		T = 4 °C		T = 25 °C	
	[CA] (mmol/L)	SD (mmol/L)	[CA] (mmol/L)	SD (mmol/L)	[CA] (mmol/L)	SD (mmol/L)	[CA] (mmol/L)	SD (mmol/L)
0.0	0.636	0.001	0.636	0.002	0.636	0.006	0.636	0.006
3.1	0.632	0.006	0.603	0.003	0.594	0.009	0.574	0.006
5.4	0.630	0.008	0.584	0.003	0.573	0.009	0.551	0.007
18.3	0.625	0.002	0.545	0.006	0.514	0.007	0.478	0.009
31.0	0.623	0.008	0.527	0.005	0.484	0.005	0.430	0.010
42.1	0.623	0.008	0.513	0.005	0.461	0.009	0.410	0.007

**Table 2:** Mean CA concentrations for samples of CA_o_ = 0.329 mmol/L (65.5 mg/L) conserved at −80, −20, 4 and 25 °C, and quantified at different times.

Time (h)	T = −80 °C		T = −20 °C		T = 4 °C		T = 25 °C	
	[CA] (mmol/L)	SD (mmol/L)	[CA] (mmol/L)	SD (mmol/L)	[CA] (mmol/L)	SD (mmol/L)	[CA] (mmol/L)	SD (mmol/L)
0.0	0.329	0.003	0.329	0.003	0.329	0.003	0.329	0.003
3.1	0.327	0.004	0.314	0.003	0.303	0.004	0.295	0.002
5.4	0.328	0.004	0.306	0.003	0.289	0.004	0.278	0.002
18.3	0.323	0.003	0.290	0.004	0.276	0.003	0.259	0.004
31.0	0.322	0.004	0.279	0.004	0.264	0.003	0.242	0.003
42.1	0.322	0.005	0.270	0.003	0.254	0.005	0.233	0.002

**Table 3:** Mean CA concentrations for samples of CA_o_ = 0.127 mmol/L (25.3 mg/L) conserved at −80, −20, 4 and 25 °C, and quantified at different times.

Time (h)	T = −80 °C		T = −20 °C		T = 4 °C		T = 25 °C	
	[CA] (mmol/L)	SD (mmol/L)	[CA] (mmol/L)	SD (mmol/L)	[CA] (mmol/L)	SD (mmol/L)	[CA] (mmol/L)	SD (mmol/L)
0.0	0.127	0.002	0.127	0.006	0.127	0.006	0.127	0.006
3.1	0.126	0.007	0.120	0.008	0.118	0.008	0.116	0.006
5.4	0.126	0.006	0.118	0.008	0.115	0.004	0.111	0.005
18.3	0.125	0.005	0.110	0.009	0.106	0.006	0.100	0.007
31.0	0.125	0.007	0.108	0.007	0.103	0.009	0.097	0.005
42.1	0.124	0.010	0.107	0.010	0.102	0.010	0.095	0.007

**Table 4:** Mean CA concentrations for samples of CA_o_ = 0.082 mmol/L (16.3 mg/L) conserved at −80, −20, 4 and 25 °C, and quantified at different times.

Time (h)	T = −80 °C		T = −20 °C		T = 4 °C		T = 25 °C	
	[CA] (mmol/L)	SD (mmol/L)	[CA] (mmol/L)	SD (mmol/L)	[CA] (mmol/L)	SD (mmol/L)	[CA] (mmol/L)	SD (mmol/L)
0.0	0.082	0.008	0.082	0.006	0.082	0.006	0.082	0.006
3.1	0.082	0.003	0.078	0.006	0.077	0.001	0.075	0.008
5.4	0.081	0.008	0.076	0.008	0.074	0.007	0.072	0.004
18.3	0.081	0.002	0.071	0.009	0.069	0.010	0.065	0.004
31.0	0.080	0.005	0.070	0.009	0.067	0.002	0.063	0.007
42.1	0.080	0.008	0.070	0.008	0.066	0.005	0.062	0.010

**Table 5:** Mean CAI concentrations of samples conserved at 4 °C and quantified at different time points.

[CAI_o_] = 0.310 mmol/L			[CAI_o_] = 0.636 mmol/L		
Time (h)	[CAI] (mmol/L)	SD (mmol/L)	Time (h)	[CAI] (mmol/L)	SD (mmol/L)
0.0	0.307	0.004	0.000	0.636	0.002
21.0	0.186	0.004	24.200	0.386	0.007
30.3	0.162	0.010	34.600	0.343	0.009
41.0	0.135	0.009	46.000	0.254	0.002

**Table 6:** Mean CAI concentrations of samples conserved at 25 °C and quantified at different timepoints.

[CAI_o_] = 0.310 mmol/L			[CAI_o_] = 0.636 mmol/L		
Time (h)	[CAI] (mmol/L)	SD (mmol/L)	Time (h)	[CAI] (mmol/L)	SD (mmol/L)
0.0	0.310	0.008	0.000	0.636	0.005
3.0	0.279	0.005	4.900	0.527	0.003
15.3	0.174	0.008	21.700	0.280	0.002
23.7	0.141	0.005	25.700	0.235	0.004
33.6	0.088	0.006	41.500	0.133	0.008
40.1	0.066	0.007	45.400	0.113	0.003

## 2 Experimental design, materials, and methods

Batch fermentations of *S. clavuligerus* DSM 41826 were carried out in a 15 L stirred tank bioreactor (Techfors S, Infors AG, Bottmingen, Switzerland) operated at 5 L filling volume using chemically defined media, pH 6.8, 0.6 vvm and 28 °C [2], [3]. Samples (50 mL) of fermentation broths were withdrawn at 36 h of cultivation coinciding with phosphate limitation and onset of the exponential growth phase. Biomass was separated by centrifugation at 12000 rpm and filtration using 0.2 μm pore size filters. Supernatants containing CA were adjusted to pH 6.8 and then vortexed and divided into 2 mL aliquots in Eppendorf tubes, according to the number of treatments. Dilutions (1:2 and 1:5) were also prepared. Finally, samples were divided into four groups and stored at the corresponding exposition temperatures (−80 °C, −20 °C, 4 °C and −25 °C).

The CA degradation was tested using a factorial experimental design, wherein concentration and temperature were defined as factors varying at three and four levels, respectively. CA_o_ of the supernatant was set as the highest level, whereas dilutions 1:2 and 1:5 were set as the medium and low levels, respectively. Twelve experimental runs were performed by duplicate. Supernatant samples were stored at −80 °C, −20 °C, 4 °C and 25 °C, respectively, for 43 h. Samples were withdrawn at 3.1 h, 5.4 h, 18.3 h, 31.0 h and 42.1 h of storage, derivatized with imidazole solution 20% m/V during 30 min at 30 °C and 800 rpm in mixing block, and immediately analyzed in a High Performance Liquid Chromatography (HPLC) device (1200 Series, Agilent Technologies, Waldbronn, Germany). Additional runs of supernatant samples with higher CA_o_ (0.636 mmol/L), from a different batch cultivation produced under identical conditions, were exposed to the above-mentioned temperatures and treated as previously indicated. In the study of stability of the chromophore CAI, a 2-squared factorial design with duplicates was applied. Derivatized CA samples with a CAI_o_ of 0.636 mmol/L and 0.310 mmol/L were stored at 4 °C and 25 °C. Aliquots were withdrawn at different intervals in a time span of 46 h.

CA is poorly retained in C-18 reverse phase columns, thus the preparation of the chromophore CAI is required for detection and quantification [1]. Derivatization reagent was prepared by dissolving 8.25 g of imidazole in 24 mL of distilled water; the pH of the derivatization reagent was adjusted to 6.8 by addition of HCl (25%v/v) and distilled water was added up to 40 mL. The derivatization of CA samples was performed by adding 100 μL of imidazole reagent to 300 μL of sample, followed by agitation in a mixing block at 800 rpm, at 30 °C during 30 min.

The derivatized samples were analyzed in an Agilent Technologies 1200 Series HPLC system (Agilent Technologies, Waldbronn, Germany) equipped with DAD detector, using a Zorbax Eclipse XDB-C-18 chromatographic column (Agilent Technologies, Waldbronn, Germany) and a C-18 guard column (Phenomenex^®^, Aschaffenburg, Germany). Quantifications were performed at 30 °C, flow rate of 1 mL/min and an injection volume of 25 μL. The mobile phase consisted of KH_2_PO_4_ (pH 3.2; 50 mM) and methanol (HPLC grade) as the solvents A and B, respectively. For the analysis, a gradient method was used as described: linear gradient from 6% to 7.6% solvent B for 8 min, linear gradient to 95% solvent B for 2 min, 95% solvent B for 2 min and linear gradient to 6% solvent B for 2 min [1]. The chromophore CAI was detected at 311 nm wavelength. The calibration line used in the quantifications is presented in Eq. (1), where [CAI] is the concentration of the chromophore detected and A is the integration area [1]. The calibration was valid in the range 0.2–400 mg/L.

(1) [CAI]=86.73A+155.86

The mechanistic approach of CA degradation included an equilibrium reaction, in which an active intermediate (I*) is produced by a first-order reversible reaction (Eqs. (2), (3))) with forward and backward constants k_1_ and k_-1_, respectively. This reaction is followed by a first-order irreversible reaction (Eq. (4)) with kinetic rate constant k_2_. In this irreversible step, the formed intermediate I* reacts with a CA molecule to form the final degradation product (D), as follows [4], [5], [6], [7]:

(2) $$\text{CA}⇆{\text{I}}^{*}\;\;\;\;\;{\text{r}}_{1}={\text{k}}_{1}\left[\text{CA}\right]–k_{-1}\left[{\text{I}}^{*}\right]\;$$

(3) $${\text{K}}_{eq}=\frac{{\;\text{k}}_{1}}{k_{-1}}$$

(4) $${\text{I}}^{*}+\text{CA}\to \text{D}\;\;\;\;\;\;\;{\text{r}}_{2}={\text{k}}_{2}\left[\text{CA}\right]$$

Numerical simulation of the reaction network was performed by solving the resulting differential equations according to the rate expressions presented in Eqs. (2), (4)) and the equilibrium constant defined by Eq. (3). The values of the kinetic rate constants are available in the literature [5]. The ordinary differential equation (ODE) system was solved by using the deterministic method LSODA for stiff and non-stiff ODEs [8].
